# The mechanisms involved in the resistance of estrogen receptor-positive breast cancer cells to palbociclib are multiple and change over time

**DOI:** 10.1007/s00432-021-03722-3

**Published:** 2021-07-09

**Authors:** Mayu Ono, Takaaki Oba, Tomohiro Shibata, Ken-ichi Ito

**Affiliations:** Division of Breast and Endocrine Surgery, Department of Surgery, Shinshu University School of Medicine, 3-1-1 Asahi, Matsumoto, Nagano 390-8621 Japan

**Keywords:** Breast cancer, Cyclin-dependent kinase inhibitor, Drug resistance, Palbociclib, Retinoblastoma gene

## Abstract

**Purpose:**

Cyclin-dependent kinase 4 and 6 (CDK4/6) inhibitors are widely used for the treatment of advanced estrogen receptor (ER)-positive breast cancer. To develop a treatment strategy for cancers resistant to CDK4/6 inhibitors, here, we established palbociclib-resistant sublines and analyzed their resistance mechanisms.

**Methods:**

Palbociclib-resistant sublines were established from T47D and MCF7 cells. Sensitivity to other drugs was assessed via the WST assay. Altered expression/phosphorylation of proteins related to signal transduction and cell cycle regulation was examined using western blotting. Copy number alterations and mutations in the retinoblastoma (*RB1*) gene were also analyzed.

**Results:**

Although an increase in CDK6 and decrease in retinoblastoma protein (Rb) expression/phosphorylation were commonly observed in the resistant sublines, changes in other cell cycle-related proteins were heterogeneous. Upon extended exposure to palbociclib, the expression/phosphorylation of these proteins became altered, and the long-term removal of palbociclib did not restore the Rb expression/phosphorylation patterns. Consistently a copy number decrease, as well as *RB1* mutations were detected. Moreover, although the resistant sublines exhibited cross-resistance to abemaciclib, their response to dinaciclib was the same as that of wild-type cells. Of note, the cell line exhibiting increased mTOR phosphorylation also showed a higher sensitivity to everolimus. However, the sensitivity to chemotherapeutic agents was unchanged in palbociclib-resistant sublines.

**Conclusion:**

ER-positive breast cancer cells use multiple molecular mechanisms to survive in the presence of palbociclib, suggesting that targeting activated proteins may be an effective strategy to overcome resistance. Additionally, palbociclib monotherapy induces mutations and copy number alterations in the *RB1* gene.

**Supplementary Information:**

The online version contains supplementary material available at 10.1007/s00432-021-03722-3.

## Introduction

Estrogen receptor (ER)-positive breast cancer (BC) accounts for approximately three-quarters of all BC cases. Postoperative adjuvant endocrine therapy has reduced BC mortality by approximately 40% (Flaum and Gradishar 2018; Group EBCTC 2015). However, the emergence of resistance and consequent disease recurrence is common. Various mechanisms underlying endocrine resistance have been reported, including ER loss or mutations, alterations in the ER pathway, activation of other signal transduction pathways, and deregulation of cell cycle signaling molecules (Osborne and Schiff 2011; Kuukasjarvi et al. 1996). Accordingly, molecular targeting drugs have been developed, such as mechanistic target of rapamycin (mTOR) or cyclin-dependent kinase (CDK) 4/6 inhibitors, thus diversifying the treatment strategies for patients with relapsed or metastatic ER-positive BC (Goetz et al. 2017; O'Leary et al. 2016; Baselga et al. 2012; Hortobagyi et al. 2016).

Supported by the results of clinical trials, three CDK4/6 inhibitors, palbociclib, ribociclib, and abemaciclib, are currently available (Goetz et al. 2017; Hortobagyi et al. 2016; Finn et al. 2016). However, most tumors that initially respond to CDK4/6 inhibitors subsequently develop resistance. Several CDK4/6 inhibitors’ resistance mechanisms have been characterized in BC cells, including the loss of or mutations in the retinoblastoma (*RB1*) gene, the alteration of CDK4/6 and CDK2 signaling, and the activation of growth signaling pathways (Klein et al. 2018; McCartney et al. 2019; Pandey et al. 2019; Portman et al. 2018). Nonetheless, the identification of biomarkers that allow the selection of appropriate strategies to treat CDK4/6 inhibitors-resistant BC is still underway (Schoninger and Blain 2020).

Herein, we established palbociclib-resistant sublines of two different ER-positive BC cell lines to elucidate the mechanism underlying palbociclib resistance. We demonstrate that the continuous exposure of ER-positive BC cells to palbociclib leads to acquired resistance via multiple mechanisms that change over time.

## Methods

### Cell culture and reagents

MCF7 and T47D cells were purchased from the American Type Cell Collection (Manassas, VA, USA). Palbociclib-resistant sublines were established via continuous exposure to a constant concentration of palbociclib for more than 6 months as described in the Supplementary Methods. Three representative clones (MCF7-P1, MCF7-P2, and T47D-PR) were used in this study. Eribulin was provided by Eisai Co., Ltd. (Tokyo, Japan). Abemaciclib, dinaciclib, and everolimus were purchased from Selleck Biotech Co., Ltd. (Tokyo, Japan). Palbociclib, paclitaxel, doxorubicin, and fluorouracil were purchased from Sigma-Aldrich (Saint Louis, MO, USA). This study was approved by the Medical Ethics Committee on Clinical Investigation of Shinshu University (No. 341).

### Water soluble tetrazolium salts (WST) assay

Cell growth inhibition was measured using a tetrazolium salt-based proliferation assay (WST assay; Wako Chemicals, Osaka, Japan); cell viability was determined as described previously (Oba et al. 2016; Oba and Ito 2018). Each experiment was independently performed and repeated at least three times.

### Western blotting

Proteins were extracted as previously described and used for western blot analyses (10 µg/lane) (Fujita et al. 2005; Ito et al. 2012). The membranes were probed with different antibodies (Supplementary Table 1). Each experiment was repeated at least three times. The protein levels were quantified using the ChemiDoc XRS (Bio-Rad Laboratories, Tokyo, Japan).

### Targeted enrichment and sequencing

The coding regions and exon/intron junctions of 93 oncogenes were enriched by multiplex PCR using the QIAseq Targeted DNA Human Breast Cancer Panel (DHS-001Z; QIAGEN, Valencia, CA, USA). The PCR products were sequenced using NextSeq 500 (Illumina, San Diego, CA, USA).

### Alignment, somatic variant calling, and copy number variation analysis

Alignment, somatic variant calling, and copy number variation (CNV) analysis were performed using the QIAGEN Data Analysis Center web service (https://ngsdataanalysis.qiagen.com/QIAseqDNA) (Xu et al. 2019). Data were visualized using the Integrative Genomics Viewer (https://software.broadinstitute.org/software/igv/). The nonsense *RB1* variant identified via the cancer panel was confirmed via Sanger sequencing; data were analyzed using the Sequence Scanner software version 1.0 (Thermo Fisher Scientific, Waltham, MA, USA).

### Multiplex ligation-dependent probe amplification (MLPA)

To confirm large deletions in *RB1*, MLPA analysis was performed using the SALSA MLPA probemix P047-E1 RB1 kit (MRC-Holland, Amsterdam, the Netherlands). The processed generated by the Applied Biosystems 3130xl Genetic Analyzer were analyzed using the Coffalyser.Net software (MRC-Holland).

### Statistical analysis

The levels of protein expression or phosphorylation obtained in the densitometric analysis of at least three independent western blots were statistically examined by two-tailed unpaired *t*-test. Statistical significance between wild-type and palbociclib-treated/resistant cells was assessed. *p* < 0.05 was considered statistically significant GraphPad Prism V.9.02 (GraphPad Software, San Diego, CA, USA).

## Results

### Establishment of palbociclib-resistant BC sublines

**Fig. 1:**
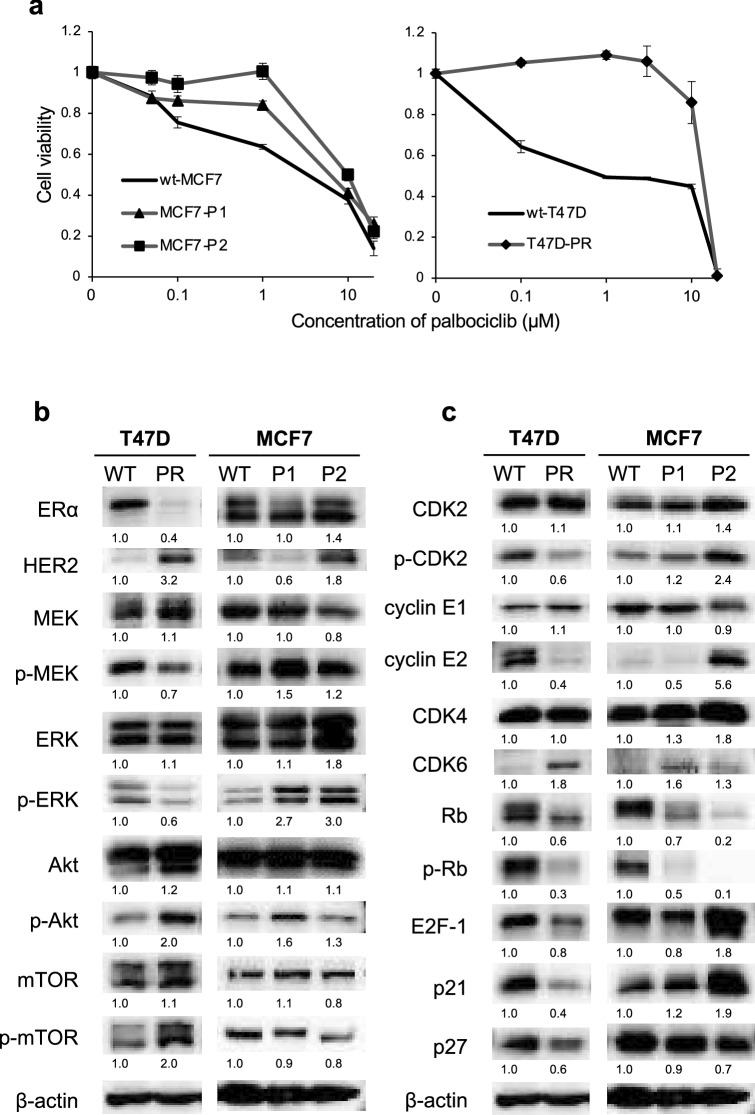
Sensitivity of palbociclib-resistant breast cancer sublines and the corresponding wild-type cells to palbociclib and protein expression profiles. **a** The sensitivity of wild-type (wt) and palbociclib-resistant breast cancer cells to palbociclib was determined using the WST assay. The black lines indicate wild-type cells (wt-MFF7, wt-T47D), and the gray lines with markers (▲, ■, ◆) indicate palbociclib-resistant sublines. Error bars represent the standard error of the values obtained in three independent experiments. **b** Expression and phosphorylation levels of the indicated proteins in palbociclib-resistant sublines (T47D-PR, MCF7-P1, and MCF7-P2) 1 month after establishment and in the corresponding wild-type cells (wt-MFF7, wt-T47D). **c** Expression of the indicated proteins in palbociclib-resistant sublines (T47D-PR, MCF7-P1, and MCF7-P2) 1 month after establishment and in the wild-type cells (wt-MFF7, wt-T47D). The proteins were detected using western blotting. β-actin was used as a loading control. The relative densitometry values (β-actin-corrected) are disclosed below each protein band. Quantitative data (relative expression levels) from three independent experiments are presented in Supplementary Fig. 1

The palbociclib-resistant MCF7-P1, MCF7-P2, and T47D-PR sublines were established in the presence of 1 µM, 2 µM, and 3 µM palbociclib, respectively (Fig. 1a). The palbociclib IC_50_ values in the context of wild-type and palbociclib-resistant cells are shown in Table 1. As demonstrated, MCF7-P1 and MCF7-P2 exhibited over 2.1- and 3.3-fold higher palbociclib resistance than wild-type MCF7 (wt-MCF7) cells, respectively. T47D-PR showed 7.9-fold higher palbociclib resistance than wild-type T47D (wt-T47D) cells.

**Table 1:** IC_50_ values for palbociclib, abemaciclib, and everolimus for wild-type and palbociclib-resistant sublines

Cell line	Molecular-targeted agents					
	Palbociclib		Abemaciclib		Everolimus	
	IC_50_ (μM)^a^	RR ratio^b^	IC_50_ (μM)^a^	RR ratio^b^	IC_50_ (nM)^a^	RR ratio^b^
MCF7	3.60 ± 0.20	–	0.36 ± 0.10	–	0.35 ± 0.10	–
MCF7-P1	7.50 ± 1.80	2.10	0.78 ± 0.20	2.20	0.40 ± 0.10	1.10
MCF7-P2	12.00 ± 2.40	3.30	2.77 ± 0.50	7.70	0.35 ± 0.20	1.00
T47D	1.70 ± 1.30	–	0.21 ± 0.10	–	4.19 ± 2.50	–
T47D-PR	13.50 ± 0.30	7.90	13.60 ± 1.40	64.80	0.74 ± 0.30	0.20

*IC*_*50*_ half-maximal inhibitory concentration

^a^Mean ± standard deviation

^b^Relative resistance ratio = IC_50_ of palbociclib-resistant cells/IC_50_ of wild-type cells

### Expression of ERα, HER2, and signal transduction pathways-related proteins in palbociclib-resistant BC cells

Next, we compared the expression or phosphorylation of signaling molecules in wild-type versus 1-month-old post establishment palbociclib-resistant cells (Fig. 1b). An increase in the expression of HER2 and in the phosphorylation of AKT and mTOR, together with a decrease in the expression of ERα were observed in T47D-PR cells. Additionally, in MCF-P1 cells, a decrease in the expression of HER2 and an increase in the phosphorylation of MEK, ERK, and AKT were observed, while in MCF-P2 cells, the expression of HER2 and the phosphorylation of ERK were increased. Of note, the average and standard error of the relative expression levels calculated from three independent experiments (referring to the represented western blot results in Fig. 1b) are presented in Supplementary Fig. 1. Altogether, these results indicate that different alterations in signal transduction pathways are induced during palbociclib resistance acquisition in BC cells.

### Expression of proteins related to cell cycle regulation in palbociclib-resistant BC sublines

We also investigated whether the expression or phosphorylation of molecules related to cell cycle regulation was altered in palbociclib-resistant sublines, 1 month post-establishment (Fig. 1c). In T47D-PR cells, the expression of CDK6 increased, whereas that of cyclin E2, Rb, E2F-1, p21, and p27 decreased; additionally, the phosphorylation of CDK2 and Rb also decreased. MCF7-P1 and MCF7-P2 exhibited unique alterations. The expression of CDK4 and CDK6 increased in both sublines; however, increases in the phosphorylation of CDK2 and in the expression of cyclin E2, E2F-1, and p21 were observed only in MCF7-P2 cells. Additionally, although the expression and phosphorylation of Rb decreased in both sublines, near-complete loss of Rb phosphorylation was only observed in MCF7-P2 cells. Overall, although a decrease in the expression and phosphorylation of Rb was commonly observed in palbociclib-resistant sublines, the alterations in molecules related to cell cycle regulation were heterogeneous.

### Time-course analysis of protein expression in MCF7 and T47D cells after short exposure to palbociclib

**Fig. 2:**
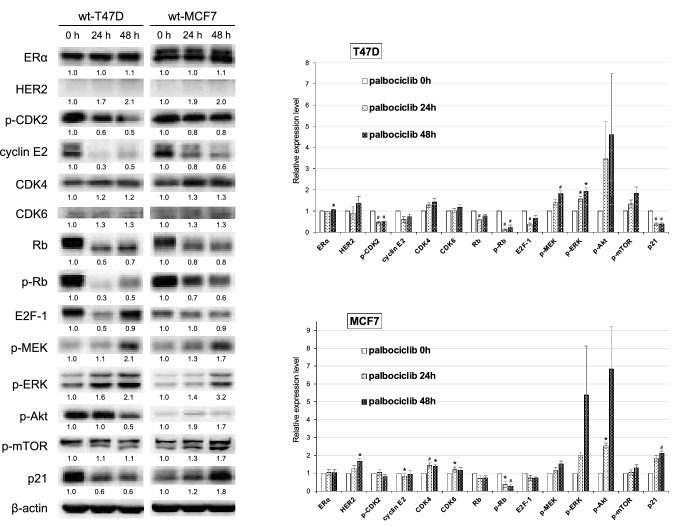
Protein expression time-course analysis after the short exposure of wild-type T47D and MCF7 cells to palbociclib. The expression of the indicated proteins in wt-T47D and wt-MCF7 cells 0, 24, and 48 h after exposure to palbociclib (2 µM for T47D, 4 µM for MCF7) was analyzed using western blotting. β-actin was used as a loading control. Representative image of western blots (*left*). The relative densitometry values (β-actin-corrected) are disclosed below each protein band. Quantitative data (relative expression levels) from three independent experiments are presented in the right panel (*p < 0.05, ^**#**^*p* < 0.01)

To examine whether the altered protein expression and phosphorylation profiles of palbociclib-resistant sublines differed from those induced by short exposure to palbociclib, we also analyzed wt-MCF7 and wt-T47D cells exposed to palbociclib (concentration near the IC_50_) for 24 or 48 h (Fig. 2). The expression of both ERα and HER2 was unchanged; however, the phosphorylation of AKT decreased 48 h after exposure to 2 µM palbociclib in wt-T47D cells. The expression of E2F-1 and cyclin E2, or of cyclin E2 alone was also reduced in wt-T47D or wt-MCF7 cells, respectively, while a slight increase in the expression of both CDK4 and CDK6 was detected in both cell lines after 48 h of palbociclib exposure. Additionally, the phosphorylation of Rb at the CDK4/6 specific site (Ser 780) decreased after 24 h of exposure of both cell lines to palbociclib; however, the levels remained higher than those detected in the counterpart-resistant sublines. Thus, these results show that the alterations induced by short-time exposure of wild-type cells to palbociclib differ from those observed in palbociclib-resistant sublines.

### Altered protein expression in palbociclib-resistant BC sublines after long-term palbociclib exposure

**Fig. 3:**
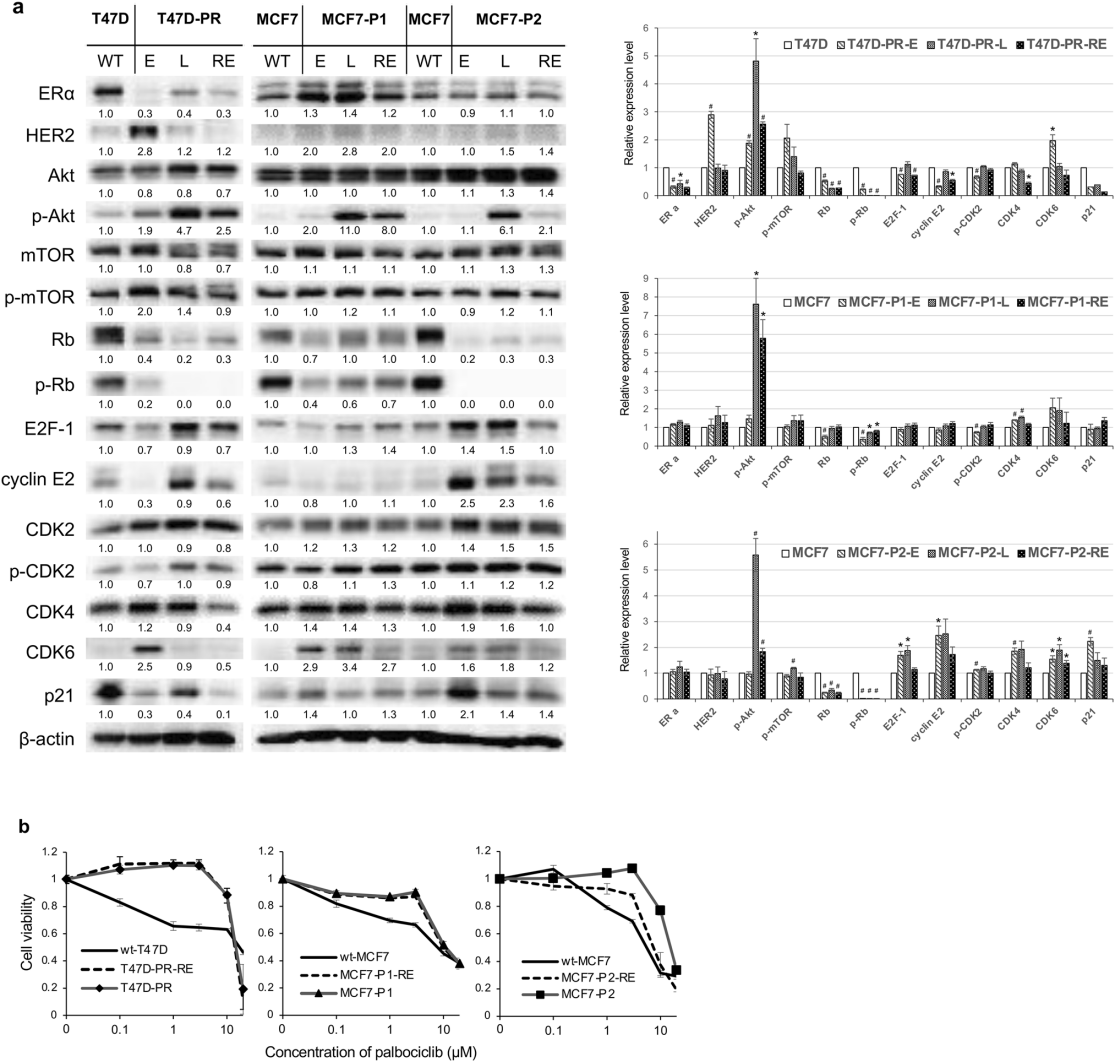
Expression of protein profiles and palbociclib sensitivity in palbociclib-resistant breast cancer sublines after long-term exposure to palbociclib or removal of palbociclib. **a** Protein expression profiles in palbociclib-resistant breast cancer sublines after long-term exposure to palbociclib or removal of palbociclib. WT: T47D and MCF7 wild-type cells. E: palbociclib-resistant sublines 1 month after establishment. L: palbociclib-resistant sublines cultured with palbociclib for 11 months. RE: palbociclib-resistant cells after removal of palbociclib for more than 3 months. β-actin was used as a loading control. Representative image of western blots (*left*). The relative densitometry values (β-actin-corrected) are disclosed below each protein band. Quantitative data (relative expression levels) from three independent experiments are presented in the right panel (*p < 0.05, ^**#**^*p* < 0.01). **b** Sensitivity of palbociclib-resistant sublines after long-term removal of palbociclib (T47D-PR-RE, MCF7-P1-RE, and MCF7-P2-RE) as compared to that of the corresponding wild-type (wt) and palbociclib-resistant sublines maintained with palbociclib (T47D-PR, MCF7-P1, and MCF7-P2). The black lines indicate wild-type cells, and the gray lines with markers (▲, ■, ◆) indicate palbociclib-resistant cells. The dotted lines indicate palbociclib-resistant cells after long-term removal of palbociclib. Error bars represent the standard error of the value obtained from triplicate experiments

Next, we compared the expression/phosphorylation of signal transduction and cell cycle regulatory proteins in palbociclib-resistant sublines exposed to palbociclib for 1 (designated as “-E”) or 11 (designated as “-L”) months (Fig. 3a).

In T47D-PR cells, the expression of ERα first decreased (T47D-PR-E cells), then increased to a level lower than that in wild-type cells after 11 months (T47D-PR-L cells). Conversely, the expression of HER2 and CDK6 increased after 1 month and decreased after 11 months. The phosphorylation of AKT and mTOR was increased in T47D-PR-E versus wild-type cells; thereafter, the phosphorylation of AKT further increased (T47D-PR-L cells). Meanwhile, the expression and phosphorylation of Rb decreased over time, and phosphorylated Rb was not detected in T47D-PR-L cells. Additionally, the expression of E2F-1 and cyclin E2 and the phosphorylation of CDK2 were increased in T47D-PR-L versus T47D-PR-E cells. With respect to the MCF7-P1 and MCF7-P2 sublines, the expression and phosphorylation of Rb was downregulated after 1 month and continued to decrease thereafter (MCF7-P1-E, and MCF7-P2-E versus MCF7-P1-L, and MCF7-P2-L cells, respectively). Additionally, the increased expression of cyclin E2, CDK2, CDK4, and E2F-1 was observed in both MCF7-P2-E and MCF7-P2-L cells, while the increase in the phosphorylation of Akt was only remarkable 11 months post-treatment (both MCF7-P1-L and MCF7-P2-L cells). Thus, these results suggest that the expression/phosphorylation patterns of these molecules are altered over time, even after the acquisition of palbociclib resistance.

To further examine how the acquisition of palbociclib-resistance affects major protein kinase signaling pathways in T47D and MCF7 cells, we compared the phosphorylation status of a panel of human kinases in wild-type cells versus T47D-PR-L, MCF7-P1-L, and MCF7-P2-L sublines (Supplementary Fig. 2). In T47D-PR-L cells, the phosphorylation of AKT, signal transducer and activator of transcription (STAT) 2/3, EGFR, SRC, and cyclic AMP response element-binding protein (CREB) was increased. On the other hand, the phosphorylation of p53, c-Jun, p70S6 kinase, ribosome S6 protein kinase (RSK) family, and STAT3 was commonly increased in MCF7-P1-L and P2-L cells. These results suggest that multiple pathways are activated in palbociclib-resistant cells, and that the mechanisms behind may depend on the innate characteristics of BC cells.

### Effect of long-term palbociclib removal on palbociclib-resistant BC sublines

Next, we investigated whether the long-term removal of palbociclib would restore the sensitivity of resistant sublines to palbociclib. T47D-PR, MCF7-P1, and MCF7-P2 cells were cultured without palbociclib for more than 3 months (designated as “-RE”). While T47D-PR-RE and MCF7-P1-RE cells maintained the same level of palbociclib resistance as T47D-PR and MCF7-P1 cells, MCF7-P2-RE cells showed a decreased resistance (IC_50_; 8.0 µM), which, however, remained higher than that of wt-MCF7 cells (Fig. 3b).

The protein expression and phosphorylation patterns in palbociclib-resistant sublines cultured without palbociclib for more than 3 months were also determined (Fig. 3a). In T47D-PR-RE cells, Rb phosphorylation remained markedly decreased, whereas the phosphorylation of AKT and the expression of cyclin E2, CDK4, and p21 were reduced (versus T47D-PR-L cells). In MCF7-P1-RE cells, the phosphorylation of AKT and the expression of CDK6 decreased (versus MCF7-P1-L cells). Additionally, in MCF7-P2-RE cells, the phosphorylation of Rb did not change, whereas the phosphorylation of AKT, E2F-1, cyclin E2, and CDK4 as well as the expression of CDK6 decreased (versus MCF7-P2-L cells).

### Cross-resistance of palbociclib-resistant ER-positive BC sublines to abemaciclib

**Fig. 4:**
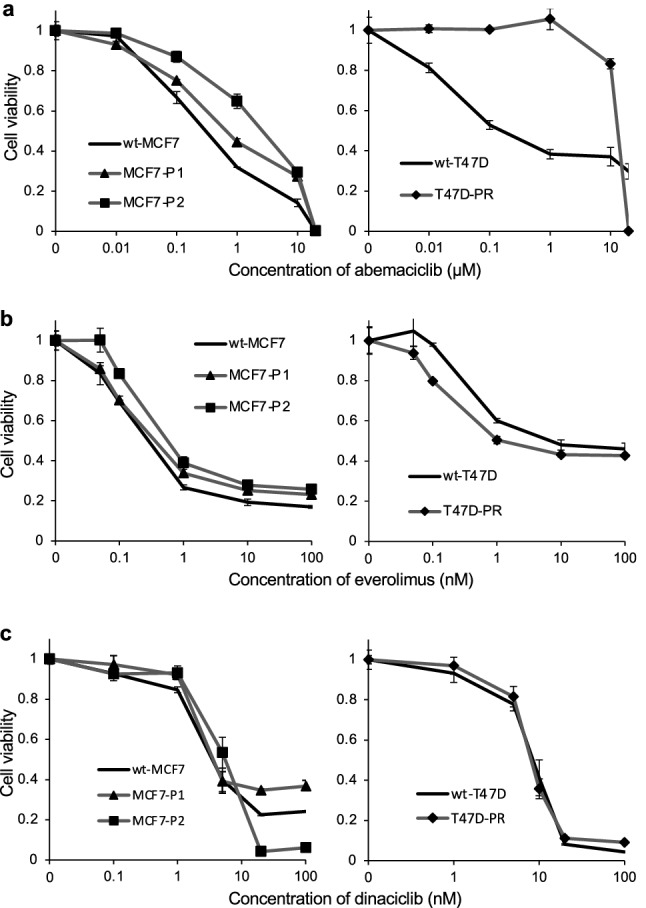
Sensitivity of palbociclib-resistant sublines to other molecular-targeted agents. The sensitivity of wild-type (wt) and palbociclib-resistant sublines (MCF7-P1, MCF7-P2, and T47D-PR) to abemaciclib, everolimus, and dinaciclib was determined using the WST assay. The black lines indicate wild-type cells, and the gray lines with markers (▲, ■, ◆) indicate palbociclib-resistant cells. Error bars represent the standard error of the value obtained from triplicate experiments. **a** Sensitivity to the CDK4/6 inhibitor, abemaciclib. **b** Sensitivity to the mTOR inhibitor, everolimus. **c** Sensitivity to the CDK1/2/5/9 inhibitor, dinaciclib

The sensitivity of T47D-PR, MCF7-P1, and MCF7-P2 cells to another CDK4/6-specific inhibitor, abemaciclib, was also determined using the WST assay (Fig. 4a). The abemaciclib IC_50_ values determined in the context of wild-type and palbociclib-resistant cells are shown in Table 1. The three palbociclib-resistant sublines demonstrated cross-resistance to abemaciclib; notably, MCF7-P2 cells, with the higher resistance levels to palbociclib were the ones that demonstrated the higher resistance to abemaciclib.

### Sensitivity of palbociclib-resistant ER-positive BC sublines to everolimus

As the phosphorylation of mTOR was increased in T47D-PR cells, next we tested whether the sensitivity to an mTOR inhibitor, everolimus, was altered in palbociclib-resistant cells (Fig. 4b). The everolimus IC_50_ in wt-T47D cells was 4.19 ± 2.5 nM, while that in T47D-PR cells was 0.74 ± 0.3 nM (Table 1). Meanwhile, no difference in sensitivity to everolimus (nor in the phosphorylation of mTOR), was observed between the wt-MCF7 and palbociclib-resistant MCF7-P1/-P2 cells. Thus, these results suggest that everolimus inhibits cell growth more efficiently in the palbociclib-resistant ER-positive sublines with elevated mTOR phosphorylation.

### Sensitivity of palbociclib-resistant ER-positive BC sublines to dinaciclib

We also investigated whether palbociclib-resistant sublines would show cross-resistance to another potent and specific CDK inhibitor (targeting CDK1, CDK2, CDK5, and CDK9), dinaciclib (Parry et al. 2010) (Fig. 4c). The results revealed that the sensitivity of the three palbociclib-resistant sublines to dinaciclib was similar to that of the corresponding wild-type cells.

### Sensitivity of palbociclib-resistant ER-positive BC sublines to various chemotherapeutic agents

**Fig. 5:**
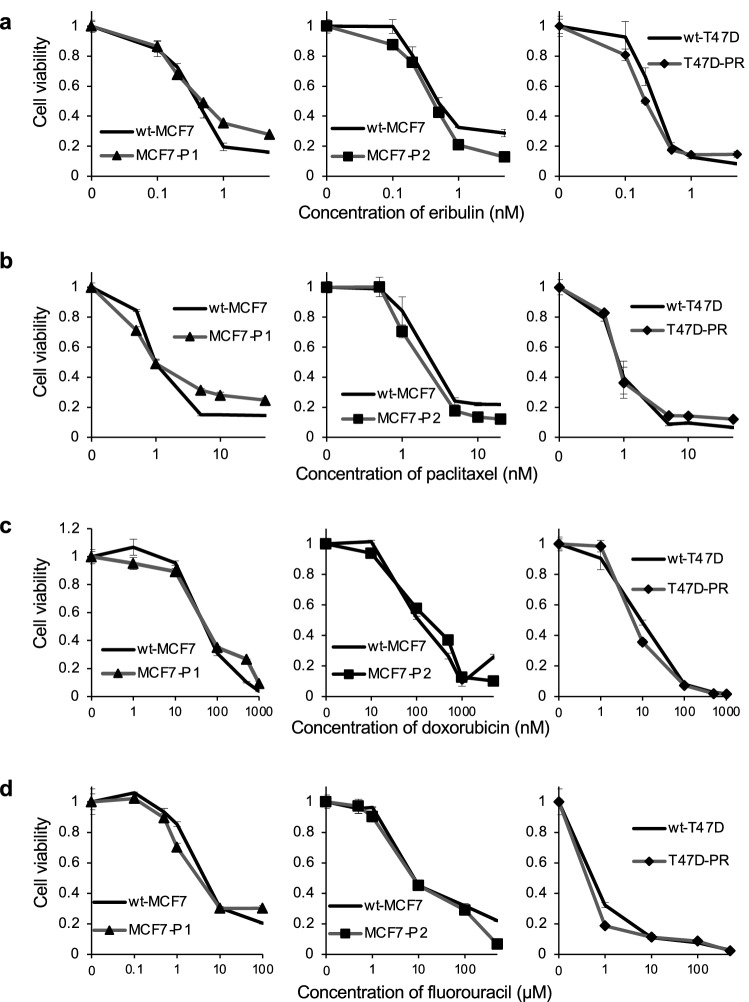
Sensitivity of palbociclib-resistant sublines to different conventional chemotherapeutic agents. The sensitivity of wild-type (wt) and palbociclib-resistant sublines (MCF7-P1, MCF7-P2, and T47D-PR) to conventional chemotherapeutic agents (paclitaxel, eribulin, doxorubicin, and fluorouracil) was determined using the WST assay. The black lines indicate wild-type cells, and the gray lines with markers (▲, ■, ◆) indicate palbociclib-resistant cells. Error bars represent the standard error of the value obtained from triplicate experiments. **a** Sensitivity to eribulin. **b** Sensitivity to paclitaxel. **c** Sensitivity to doxorubicin. **d** Sensitivity to fluorouracil

Since CDK4/6 inhibitors arrest the cell cycle, next we assessed the sensitivity of palbociclib-resistant sublines to four other chemotherapeutic agents (eribulin, paclitaxel, doxorubicin, and fluorouracil) (Fig. 5). In line with the above results, the sensitivity of palbociclib-resistant sublines to these drugs was similar compared to the wild-type cells.

### Sensitivity of palbociclib-resistant ER-positive BC sublines to the combination of palbociclib and fulvestrant

**Fig. 6:**
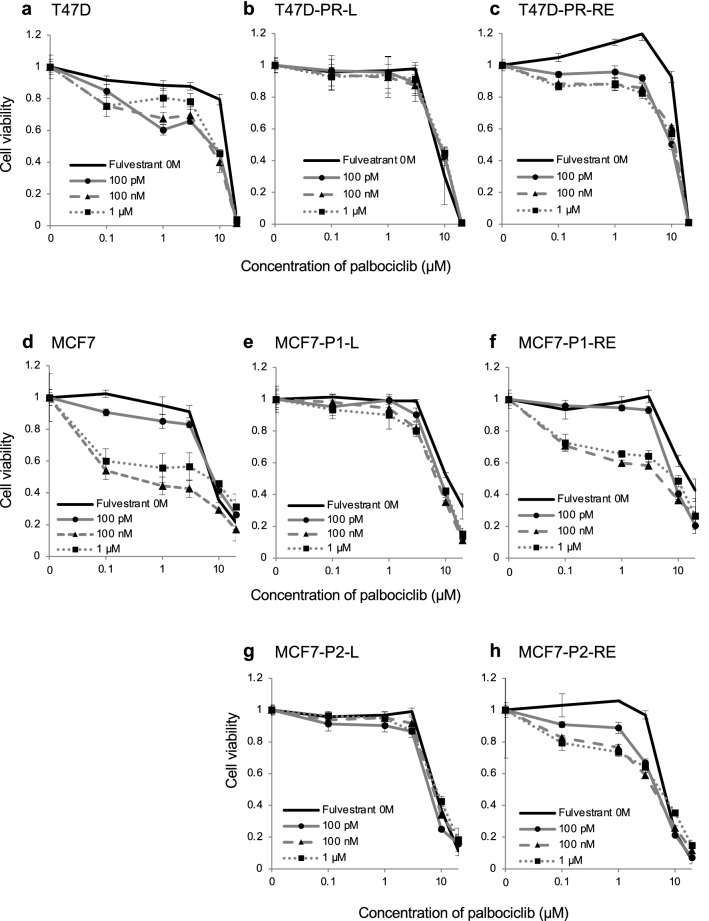
Sensitivity of palbociclib-resistant sublines to the combination of palbociclib and fulvestrant. The sensitivity of wild-type cells (**a** and **d**), palbociclib-resistant sublines maintained with palbociclib for long-term (**b**, T47D-PR-L; **e**, MCF7-P1-L; **g**, MCF7-P2-L) and palbociclib-resistant sublines deprived palbociclib for more than 3 months (**c**, T47D-PR-RE; **f**, MCF7-P1-RE; **h**, MCF7-P2-RE) to the combination therapy with palbociclib and fulvestrant was determined using the WST assay. The black lines indicate the cells without fulvestrant. The gray lines with markers (▲, ■, ◆) indicate the cells treated with palbociclib in combination with 100 pM (●), 100 nM (▲), and 1 µM (■) of fulvestrant. Error bars represent the standard error of the value obtained from triplicate experiments

Next, we assessed the response to combination therapy with palbociclib and fulvestrant in wild-type and palbociclib-resistant cells, either long-term exposed to palbociclib (T47D-PR-L, MCF7-P1-L, and MCF7-P2-L) or deprived of palbociclib for more than 3 months (T47D-PR-RE, MCF7-P1-RE, and MCF7-P2-RE) (Fig. 6). In wt-T47D and -MCF7 cells, the addition of fulvestrant enhanced the growth inhibitory effect of palbociclib. In contrast, this effect was not observed in T47D-PR-L, MCF7-P1-L, and MCF7-P2-L cells. However, after deprivation from palbociclib for more than 3 months, T47D-PR-RE, MCF7-P1-RE, and MCF7-P2-RE cells demonstrated sensitivity to fulvestrant equivalent to that of wild-type cells. Of note, the effect of fulvestrant peaked out at more than 100 nM in both wild-type and palbociclib-resistant cells.

### Mutation and copy number analyses of palbociclib-resistant BC sublines

**Fig. 7:**
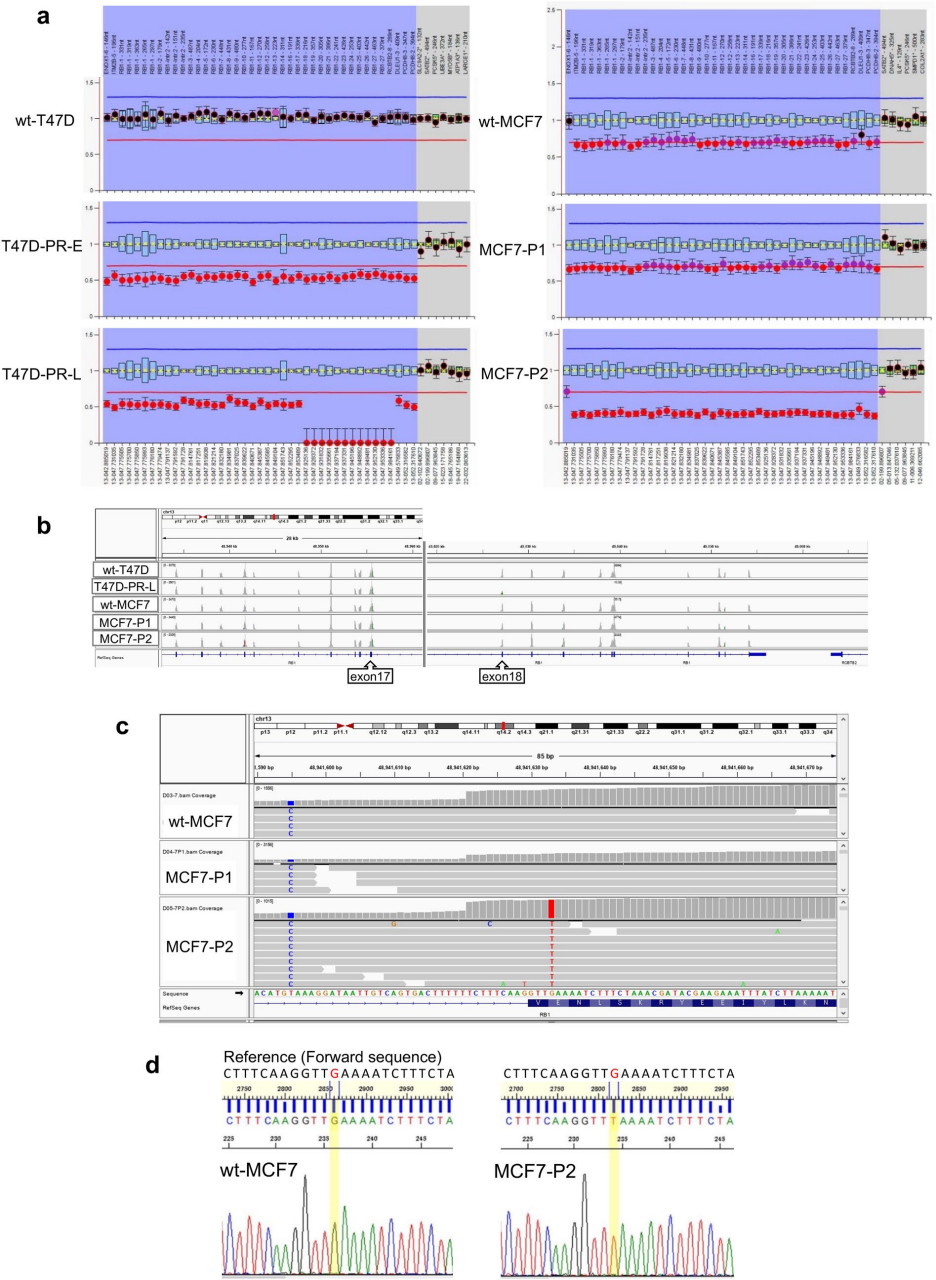
Analyses of *Rb1* mutations and copy number alterations in palbociclib-resistant sublines. **a** Analysis of *RB1* copy number alterations via Multiplex Ligation-Dependent Probe Amplification (MLPA) in wt-T47D, T47D-PR-E, T47D-PR-L, wt-MCF7, MCF7-P1, and MCF7-P2 cells. *RB1* and the flanking genes are aligned in the blue area; reference genes are aligned in the gray area of each chart. **b** Integrative Genomics Viewer (IGV) image for targeted sequencing data of *RB1* in wild-type and palbociclib-resistant T47D and MCF7 cells. In T47D-PR-L cells, a deletion after exon 18 was detected. **c** Snapshot of IGV focusing on a part of *RB1* in wt-MCF7, MCF7-P1, and MCF7-P2 cells. The red bar indicates a single nucleotide alteration in exon 10 of the *RB1* gene in MCF7-P2 cells (lower window), while no mutation in the same region was detected in wt-MCF7 (upper window) and palbociclib-resistant MCF7-P1 (middle window) cells. **d** Sanger sequencing confirmed a single nucleotide alteration from G to T (at 48941633G)

Next, we investigated whether genetic alterations in *RB1* occurred in the context of palbociclib resistance using digital DNA sequencing and MLPA analysis. In T47D-PR-E cells, the copy number of the region encompassing *RB1* was reduced to half of that in wild-type cells (Fig. 7a). Additionally, a major deletion after exon 18 of *RB1* was observed in T47D-PR-L cells maintained with palbociclib for an additional 10 months, (Fig. 7b). A decrease in the copy number of the region encompassing *RB1* (chromosome 13q14) in wt-MCF7 cells was detected in line with the previously reported studies (Fig. 7a) (Shadeo and Lam 2006; Przybytkowski et al. 2011). However, the copy number of the region encompassing *RB1* was not altered in palbociclib-resistant MCF7-P1 cells, and no mutations in *RB1* were detected. In contrast, the copy number of the region encompassing *RB1* was reduced in MCF7-P2 cells and a single-base substitution in exon 10 of *RB1* [NM_000321.2(RB1): g.48941633G>T (p.Glu315*)] predicted to create a termination codon was also observed (Fig. 7c, d). Based on these results, we inferred that mutations in *RB1* caused the irreversible loss of Rb phosphorylation observed in T47D-PR and MCF7-P2 cells after the long-term removal of palbociclib (Fig. 3a).

## Discussion

Herein, we demonstrate that ER-positive BC cells acquire resistance to palbociclib through several molecular mechanisms, including the alteration in intracellular signal transduction and cell cycle regulation systems over time (even after the acquisition of resistance). Mutations in *RB1* have been reported in the circulating tumor DNA (ctDNA) of several BC patients and in patient-derived tumor xenograft models (Herrera-Abreu et al. 2016; Condorelli et al. 2018; O'Leary et al. 2018). Of note, all *RB1* mutations reported in the clinics were derived from patients treated with a combination of endocrine therapy and CDK4/6 inhibitors (Herrera-Abreu et al. 2016; Condorelli et al. 2018; O'Leary et al. 2018). However, no studies have reported the generation of *RB1* mutations in the presence of a CDK4/6 inhibitor in cultured BC cells in vitro. Here we identified *RB1* mutations in two palbociclib-resistant ER-positive BC sublines treated with palbociclib alone. To the best of our knowledge, this is the first report demonstrating the induction of mutations in *RB1* in BC cells treated with a CDK4/6 inhibitor alone.

Past studies have demonstrated several mechanisms underlying the resistance of BC cells to CDK4/6 inhibitors, including loss of, or mutations in *RB1,* altered CDK4/6 and CDK2 signaling pathways, and activation of growth signaling pathways (McCartney et al. 2019; Portman et al. 2018; Schoninger and Blain 2020; Guarducci et al. 2018). In the present study, exposure to palbociclib downregulated the expression and phosphorylation of Rb in MCF7 and T47D cells within 48 h. Consistent with previous studies (Caldon et al. 2009, 2012), the expression of E2F-1, and its transcriptional target cyclin E2 was reduced by palbociclib in both cell lines, indicating the inhibition of cyclin E2-CDK2 complexes. However, the emerging resistance mechanisms were not uniform across sublines. Particularly, we noted for the first time that the prolonged exposure of MCF7 and T47D cells to palbociclib after resistance acquisition induced further changes in the expression and phosphorylation of related molecules.

Loss of, or mutations in Rb has been the most frequently observed change in cells resistant to CDK4/6 inhibitors regardless of the cancer type (Herrera-Abreu et al. 2016; Taylor-Harding et al. 2015; Bollard et al. 2017). Recently, Iida et al. (Iida et al. 2019) reported the restoration of sensitivity in ribociclib-resistant cells after deprivation of ribociclib. In contrast, our established palbociclib-resistant sublines maintained palbociclib resistance even after they were deprived of palbociclib for 3 months or longer, with the persistent loss of Rb phosphorylation (in T47D-PR-RE and MCF7-P2-RE cells). Further, we detected *RB1* mutations in these sublines. Of note, *RB1* mutations in the former cell line emerged during long-term exposure to palbociclib after the acquisition of resistance. Recently, Condorelli et al. (2018) reported the emergence of an *RB1* mutation in ctDNA from patients treated with a combination of endocrine therapy and CDK4/6 inhibitors for a long period. This observation, together with our data, suggests that long-term treatment with CDK4/6 inhibitors can induce *RB1* mutations in ER-positive BC cells. Interestingly, the *RB1* mutations detected in this study differed from those reported previously (Herrera-Abreu et al. 2016; Condorelli et al. 2018; O'Leary et al. 2018). Additionally, a CNV in *RB1* was detected in these palbociclib-resistant sublines, suggesting that CDK4/6 inhibitors induce various changes in *RB1.* Of note, in the present study, *RB1* CNVs and mutations were not observed in MCF7-P1 cells, those maintained at a lower concentration of palbociclib. Therefore, our results suggest that the concentration of palbociclib may influence alterations in *RB1*.

Although the Rb function was suppressed in T47D-PR, MCF7-P1, and MCF7-P2 cells, this was not the only alteration detected; there were differences in the expression/phosphorylation of other molecules involved in signal transduction and cell cycle regulation pathways. Interestingly, the established palbociclib-resistant sublines showed cross-resistance to another CDK4/6 inhibitor, abemaciclib, despite the differences in the expression/phosphorylation status of proteins. Hence, it may be necessary to administer drugs targeting molecules other than CDK4/6 to efficiently treat patients with palbociclib-resistant BC.

Additionally, fulvestrant did not show additional growth inhibitory effect in the presence of palbociclib in the context of palbociclib-resistant sublines; however, after palbociclib deprivation for 3 months, these sublines became sensitive to fulvestrant, even though they maintained palbociclib resistance. In these palbociclib-resistant sublines, the augmentation of AKT phosphorylation became statistically significant after long-term palbociclib exposure. However, long-term removal of palbociclib attenuated the phosphorylation of AKT in all three sublines. From these findings, we suggest that AKT plays an important role, either directly or indirectly, in the survival of these BC cells under pressure by palbociclib. A decrease in AKT phosphorylation may be involved in the recovery of the additional growth inhibitory effect by fulvestrant observed in the palbociclib-resistant sublines deprived of palbociclib for 3 months. However, further experiments are required to elucidate the precise mechanisms. Altogether, these findings suggest that the signal transduction pathways activated by palbociclib may alter the susceptibility of BC cells, a hypothesis that must be proved via further experiments.

The cyclin E-CDK2 complex plays a key role in the progression of the cell cycle from G1 to the S phase. Interestingly, previous studies have demonstrated that CDK4/6 inhibitor-resistant cells lose their dependence on cyclin D1-CDK4/6 signaling; they activate bypass signaling pathways to survive, including the cyclin E-CDK2, and PI3K/AKT/mTOR pathways (Caldon et al. 2012; Jansen et al. 2017). In the present study, the expression of cyclin E2 and phosphorylation of CDK2 were remarkably increased in MCF7-P2 cells. Additionally, dinaciclib, a pan-CDK inhibitor (Bose et al. 2013), showed the same growth-inhibitory effect on palbociclib-resistant and the corresponding wild-type cells. Hence, these results suggest that targeting other CDKs may be a promising strategy to overcome resistance to CDK4/6 inhibitors.

On a different note, increased mTOR phosphorylation was observed in T47D-PR cells, whose sensitivity to an mTOR inhibitor, everolimus, was increased. Recently, the potential therapeutic benefits of combinatorial treatment with CDK4/6 and PI3K/AKT/mTOR pathway inhibitors to overcome resistance to the former, have been reported (Herrera-Abreu et al. 2016; Jansen et al. 2017; Michaloglou et al. 2018; Chen et al. 2019). The effects obtained with everolimus in our study support these findings.

Meanwhile, the conventional chemotherapeutic agents continue to occupy an essential role in the treatment of advanced or metastatic ER-positive BC. Herein, our established palbociclib-resistant sublines did not show cross-resistance to the four tested representative cytotoxic chemotherapeutic agents. Although further elucidation is required, our results suggest that the prior use of CDK4/6 inhibitors do not affect the efficacy of subsequently administered traditional chemotherapeutic agents, and consequently support their use for the treatment of recurrent BC.

This study is not without limitations. First, we characterized the palbociclib-resistant mechanisms in the context of only two different cell lines. Since the characteristics of ER-positive BC cells are diverse, it is necessary to verify whether these mechanisms are transversal to other ER-positive BC cells. Moreover, it remains unclear why palbociclib affects the status of *RB1*. Also, as the results of phospho-protein kinase arrays (Supplementary Fig. 2) suggested, the involvement of other kinases such as RSK or JNK as well as of other signaling systems such as the JAK-STAT pathway, their roles in palbociclib resistance should also be elucidated. However, in line with previous preclinical and clinical data, our results clearly show that the mechanisms underlying CDK4/6 inhibitor resistance are complex and dependent on the duration of drug pressure.

## Conclusion

Here, we show that different molecular mechanisms are associated with resistance to palbociclib in BC cells. Importantly, we also show that *RB1* mutations, as well as CNV, can be induced with palbociclib alone. Of note, our data also support the notion that agents inhibiting alternative pathways must be used to treat drug-resistant BC. In the near future, the usage of CDK inhibitors is expected to become more widespread, including as adjuvant drugs. Hence, it will become more and more important to analyze the molecular status of tumors in real-time, using methods such as ctDNA analysis, to develop the best strategy to overcome drug resistance.

## Supplementary Information

Below is the link to the electronic supplementary material.

Supplementary file1 Supplementary Fig. 1. Relative protein expression levels in wild-type and palbociclib-resistant breast cancer cells (referent to Fig. 1b and c). The expression or phosphorylation of different proteins was analyzed by western blotting as presented in Fig. 1b and c. β-actin was used as an internal control in each experiment, for normalization purposes. Histograms represent the average relative protein expression (and standard error) calculated from three independent experiments. a Relative expression of proteins in T47D and T47D-PR cells. b Relative expression of proteins in MCF7, MCF7-P1 and MCF7-P2 cells (* p < 0.05, #p < 0.01). (PDF 36 KB)

Supplementary file2 Supplementary Fig. 2. Human phospho-kinase array analysis of cell lysates extracted from wild-type and palbociclib-resistant T47D and MCF7 cells. The levels of phosphorylated kinases in cell lysates (a, wt-T47D and T47D-PR-L cells; b, wt-MCF7, MCF7-P1-L, and MCF7-P2-L cells) were analyzed using the Proteome Profiler Human Phospho-Kinase Array Kit (ARY003C). The representative upregulated kinases in palbociclib-resistant sublines are indicated on the array membrane (left panel). The signal intensity of each spot was quantitated using Image Lab; relative phosphorylation was determined using the Phospho-Kinase Array analysis software. Positive fold regulation indicates a relative fold increase in palbociclib-resistant sublines compared to wild-type cells (right panel: c, T47D-PR-L vs. wt-T47D cells; d, MCF7-P1-L vs. wt-MCF7 cells; e, MCF7-P2-L vs. wt-MCF7 cells). (PDF 157 KB)

Supplementary file3 (DOCX 19 KB)

Supplementary file4 (DOCX 23 KB)

## Data Availability

The data supporting the findings of this work are available from the authors upon reasonable request.

## Abbreviations

CDK4/6: Cyclin-dependent kinase 4 and 6
CNV: Copy number variation
ER: Estrogen receptor
ERK: Extracellular signal-regulated kinase
FBS: Fetal bovine serum
HER2: Human epidermal growth factor receptor-type 2
MAPK: Mitogen-activated protein
MLPA: Multiplex ligation-dependent probe amplification
PI3K: Phosphoinositide 3-kinase
Rb: Retinoblastoma
Wt: Wild-type
RSK: Ribosome S6 protein kinase
