# Supplementary figures and images for: The mechanisms involved in the resistance of estrogen receptor-positive breast cancer cells to palbociclib are multiple and change over time
Source: J Cancer Res Clin Oncol. 2021 Jul 9;147(11):3211–24. doi: 10.1007/s00432-021-03722-3 (PMC8484193; doi:10.1007/s00432-021-03722-3)

# Supplementary fig. 1

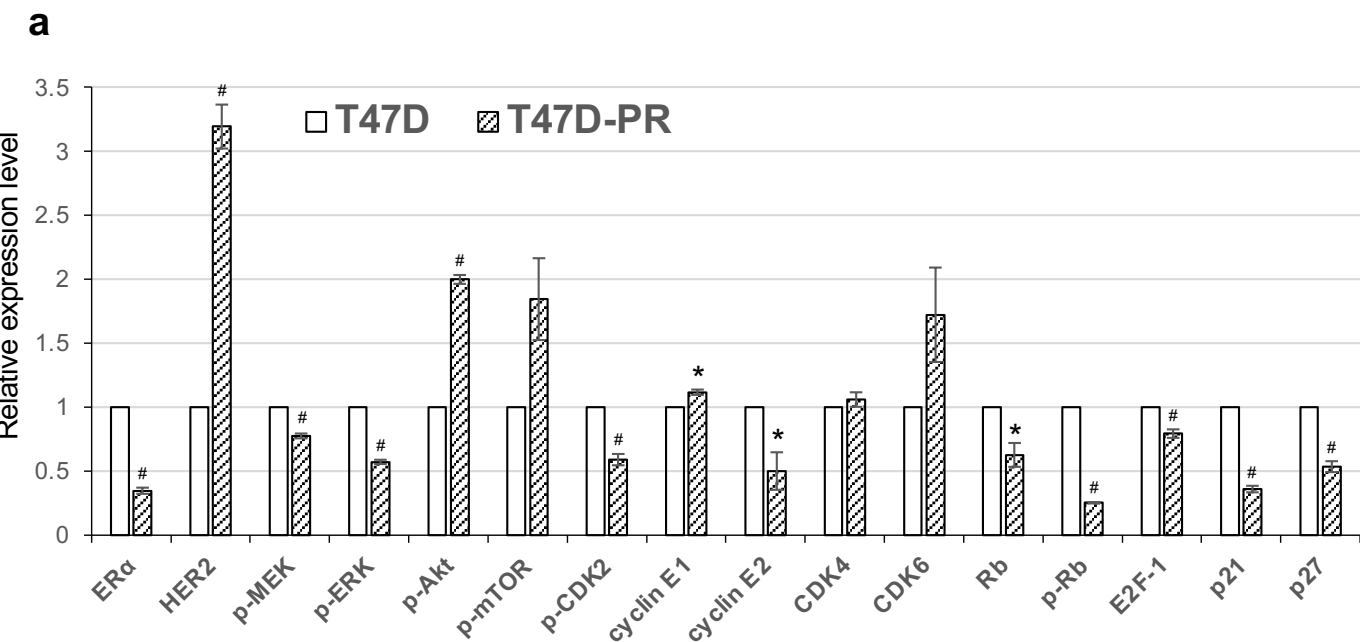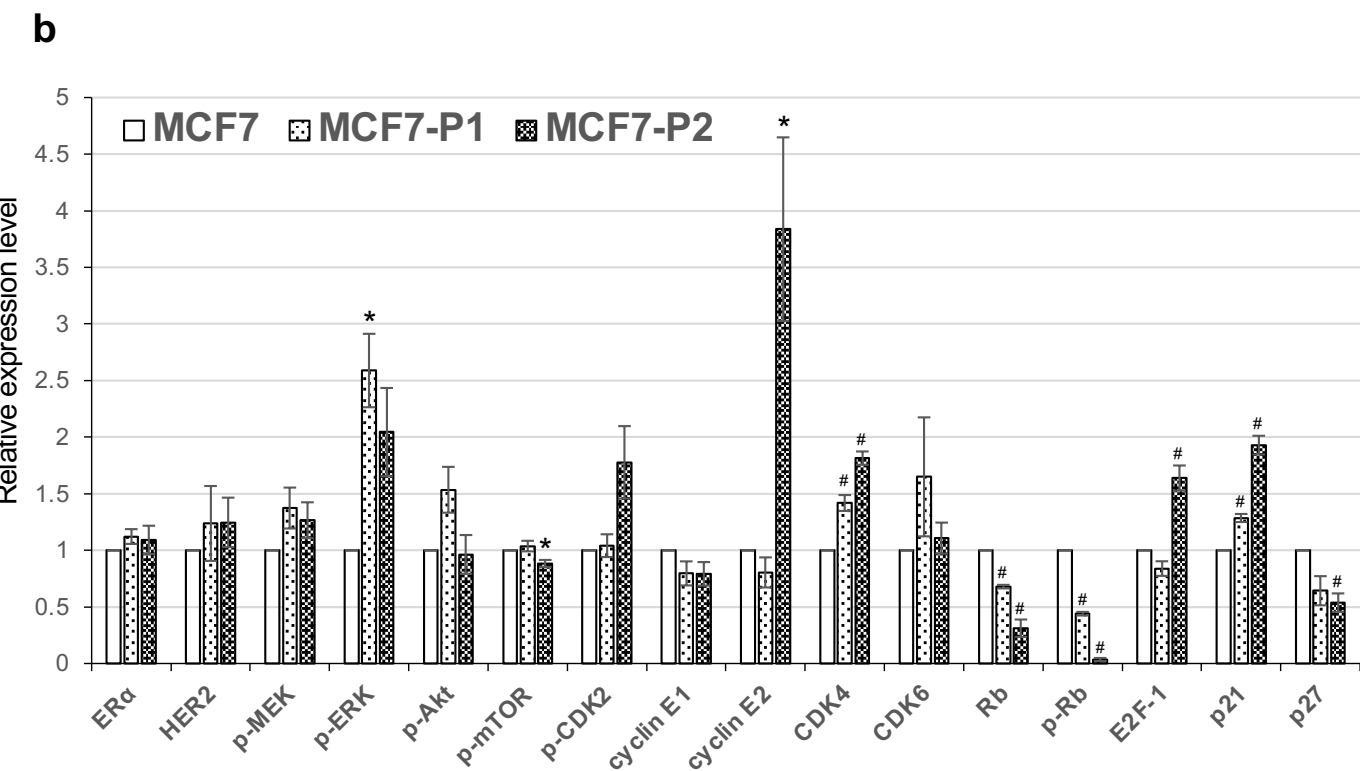

Supplement: Supplementary file 1 — Supplementary file1 Supplementary Fig. 1. Relative protein expression levels in wild-type and palbociclib-resistant breast cancer cells (referent to Fig. 1b and c). The expression or phosphorylation of different proteins was analyzed by western blotting as presented in Fig. 1b and c. β-actin was used as an internal control in each experiment, for normalization purposes. Histograms represent the average relative protein expression (and standard error) calculated from three independent experiments. a Relative expression of proteins in T47D and T47D-PR cells. b Relative expression of proteins in MCF7, MCF7-P1 and MCF7-P2 cells (* p < 0.05, #p < 0.01). (PDF 36 KB) [file 432_2021_3722_MOESM1_ESM.pdf]

## Supplementary fig. 2

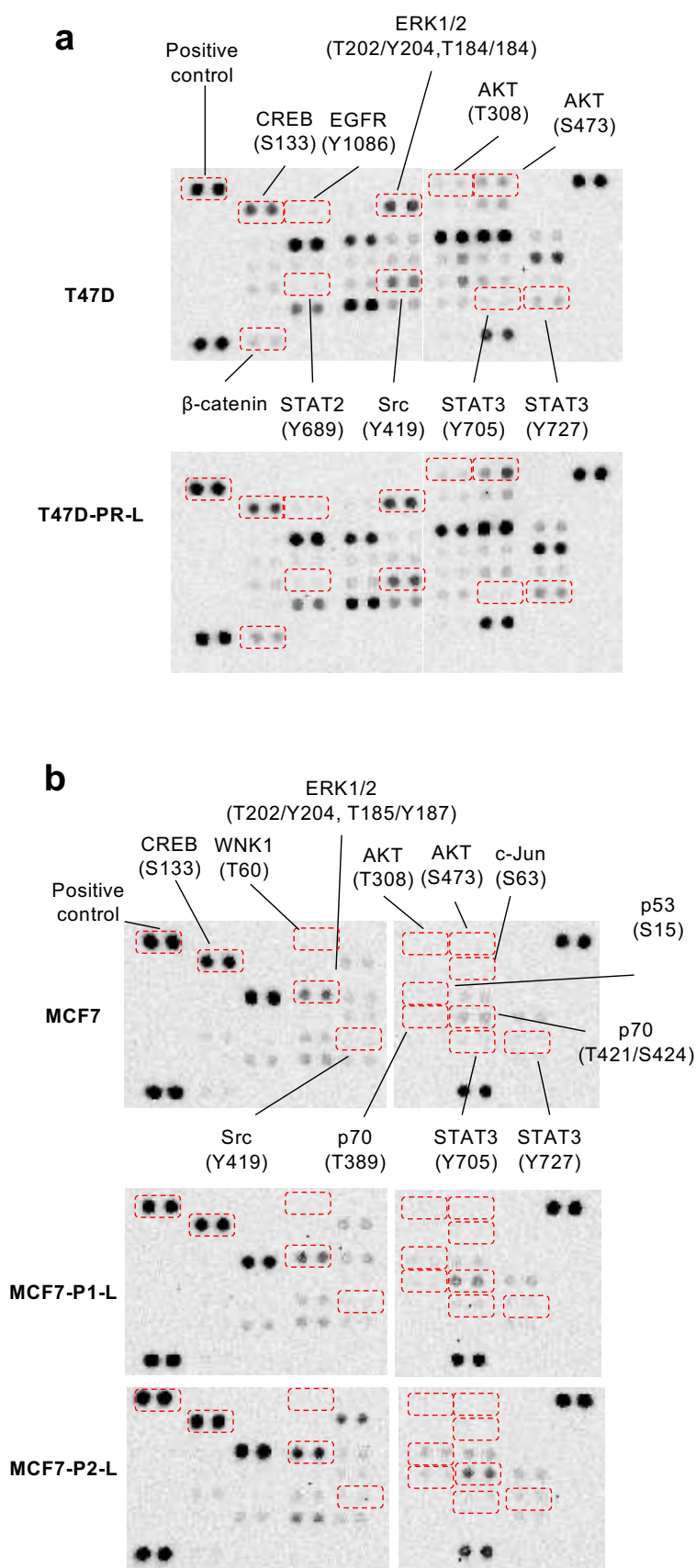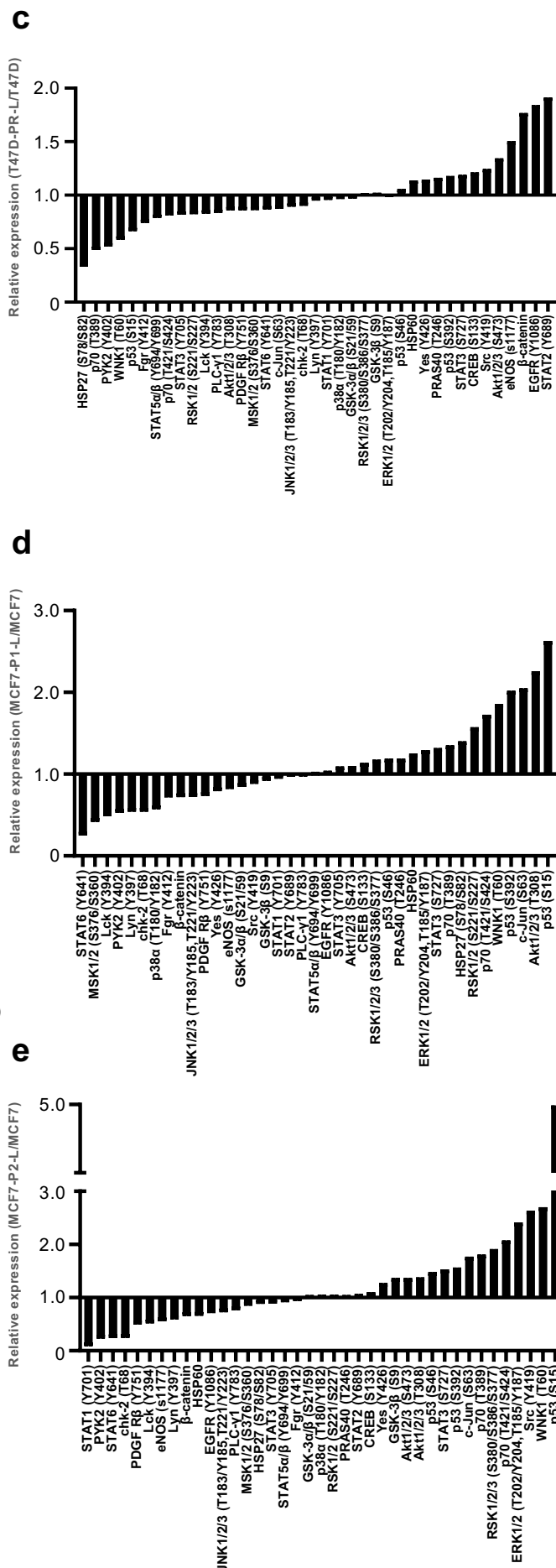

Supplement: Supplementary file 2 — Supplementary file2 Supplementary Fig. 2. Human phospho-kinase array analysis of cell lysates extracted from wild-type and palbociclib-resistant T47D and MCF7 cells. The levels of phosphorylated kinases in cell lysates (a, wt-T47D and T47D-PR-L cells; b, wt-MCF7, MCF7-P1-L, and MCF7-P2-L cells) were analyzed using the Proteome Profiler Human Phospho-Kinase Array Kit (ARY003C). The representative upregulated kinases in palbociclib-resistant sublines are indicated on the array membrane (left panel). The signal intensity of each spot was quantitated using Image Lab; relative phosphorylation was determined using the Phospho-Kinase Array analysis software. Positive fold regulation indicates a relative fold increase in palbociclib-resistant sublines compared to wild-type cells (right panel: c, T47D-PR-L vs. wt-T47D cells; d, MCF7-P1-L vs. wt-MCF7 cells; e, MCF7-P2-L vs. wt-MCF7 cells). (PDF 157 KB) [file 432_2021_3722_MOESM2_ESM.pdf]
