# Supplementary material for: The mechanisms involved in the resistance of estrogen receptor-positive breast cancer cells to palbociclib are multiple and change over time
Source: J Cancer Res Clin Oncol. 2021 Jul 9;147(11):3211–24. doi: 10.1007/s00432-021-03722-3 (PMC8484193; doi:10.1007/s00432-021-03722-3)
Supplement: Supplementary file 3 — Supplementary file3 (DOCX 19 KB) [file 432_2021_3722_MOESM3_ESM.docx]

**Journal of Cancer Research and Clinical Oncology**

**The mechanisms involved in the resistance of estrogen receptor-positive breast cancer cells to palbociclib are multiple and change over time**

Mayu Ono, Takaaki Oba, Tomohiro Shibata, and Ken-ichi Ito*

*Division of Breast and Endocrine Surgery, Department of Surgery, Shinshu University School of Medicine, 3-1-1 Asahi, Matsumoto, Japan*

***Corresponding author:**

Ken-ichi Ito

E-mail: kenito@shinshu-u.ac.jp

**Supplementary table 1. List of antibodies used for western blot analysis.**

| **Antibody** | **Company** | **Catalog number** | **Dilution** |
| --- | --- | --- | --- |
| anti-ERα | Santa Cruz Biotechnology, Heidelberg, CA, USA | SC-7207 | 1:200 |
| anti-HER2 | Cell Signaling Technology (CST), Danvers, MA, USA | 2165S | 1:1000 |
| anti-MEK | CST | 4694S | 1:1000 |
| anti-phospho-MEK (Ser217/221) | CST | 9121S | 1:1000 |
| anti-Erk | CST | 4695S | 1:1000 |
| anti-phospho Erk (Thr202/Tyr204) | CST | 4370S | 1:2000 |
| anti-Akt | CST | 4691S | 1:1000 |
| anti-phospho-Akt (Ser473) | CST | 4058S | 1:1000 |
| anti-mTOR | CST | 2972S | 1:1000 |
| anti-phospho-mTOR (Ser2448) | CST | 2971S | 1:1000 |
| anti-CDK2 | CST | 2546S | 1:1000 |
| anti-phspho-CDK2 (Thr160) | CST | 2561S | 1:1000 |
| anti-CDK4 | CST | 2790S | 1:1000 |
| anti-CDK6 | CST | 3136S | 1:2000 |
| anti-Rb | CST | 9313S | 1:1000 |
| anti-phospho-Rb (Ser780) | CST | 9307S | 1:1000 |
| anti-E2F-1 | CST | 3742S | 1:1000 |
| anti-cyclin E1 | CST | 20808S | 1:1000 |
| anti-cyclin E2 | CST | 4132S | 1:1000 |
| anti-p21 | CST | 2947S | 1:1000 |
| anti-p27 | CST | 3686S | 1:1000 |
| β-actin (loading control) | Sigma, Saint Louis, MO | A5441 | 1:5000 |
