# Supplementary material for: The mechanisms involved in the resistance of estrogen receptor-positive breast cancer cells to palbociclib are multiple and change over time
Source: J Cancer Res Clin Oncol. 2021 Jul 9;147(11):3211–24. doi: 10.1007/s00432-021-03722-3 (PMC8484193; doi:10.1007/s00432-021-03722-3)
Supplement: Supplementary file 4 — Supplementary file4 (DOCX 23 KB) [file 432_2021_3722_MOESM4_ESM.docx]

**Journal of Cancer Research and Clinical Oncology**

**The mechanisms involved in the resistance of estrogen receptor-positive breast cancer cells to palbociclib are multiple and change over time**

Mayu Ono, Takaaki Oba, Tomohiro Shibata, and Ken-ichi Ito*

*Division of Breast and Endocrine Surgery, Department of Surgery, Shinshu University School of Medicine, 3-1-1 Asahi, Matsumoto, Japan*

***Corresponding author:**

Ken-ichi Ito

E-mail: kenito@shinshu-u.ac.jp

**Supplementary Materials and Methods**

**Establishment of palbociclib-resistant breast cancer sublines**

After the maintenance of T47D cells with 3 µM of palbociclib, and of MCF7 cells with 1 or 2 µM of palbociclib for several weeks, the bulk palbociclib-resistant cell population was subjected to the limiting dilution method. Thereafter, several resistant clones were selected in the presence of the indicated concentration of palbociclib for three to four months. One representative clone was selected from the cells established in each culture condition - designated as the sublines T47D-PR, MCF7-P1, and MCF7-P2. These sublines were used in all experiments. Of note, all cells were checked regularly for *Mycoplasma* contamination.

**Phosphokinase Array**

We purchased the Proteome Profile Human Phospho-kinase Array Kit (ARY003C) from R&D Systems (Minneapolis, MN, USA) and used it to assess the phosphorylation status of wild-type and palbociclib-resistant sublines as per the manufacturer’s instructions. Briefly, total proteins (300 μg/array membrane) were incubated overnight with the Proteome Profiler Human Phospho-Kinase Array and the signal intensity of each of the generated spots was quantitated using Image Lab (Bio-Rad Laboratories, Hercules, CA, USA).
